# Comparison of Low-Pass Filters for SPECT Imaging

**DOI:** 10.1155/2020/9239753

**Published:** 2020-04-01

**Authors:** Inayatullah S. Sayed, Siti S. Ismail

**Affiliations:** Department of Diagnostic Imaging and Radiotherapy, Kulliyyah of Allied Health Sciences, International Islamic University Malaysia, Kuantan Campus, 25200 Kuantan, Pahang, Malaysia

## Abstract

In single photon emission computed tomography (SPECT) imaging, the choice of a suitable filter and its parameters for noise reduction purposes is a big challenge. Adverse effects on image quality arise if an improper filter is selected. Filtered back projection (FBP) is the most popular technique for image reconstruction in SPECT. With this technique, different types of reconstruction filters are used, such as the Butterworth and the Hamming. In this study, the effects on the quality of reconstructed images of the Butterworth filter were compared with the ones of the Hamming filter. A Philips ADAC forte gamma camera was used. A low-energy, high-resolution collimator was installed on the gamma camera. SPECT data were acquired by scanning a phantom with an insert composed of hot and cold regions. A Technetium-99m radioactive solution was homogenously mixed into the phantom. Furthermore, a symmetrical energy window (20%) centered at 140 keV was adjusted. Images were reconstructed by the FBP method. Various cutoff frequency values, namely, 0.35, 0.40, 0.45, and 0.50 cycles/cm, were selected for both filters, whereas for the Butterworth filter, the order was set at 7. Images of hot and cold regions were analyzed in terms of detectability, contrast, and signal-to-noise ratio (SNR). The findings of our study indicate that the Butterworth filter was able to expose more hot and cold regions in reconstructed images. In addition, higher contrast values were recorded, as compared to the Hamming filter. However, with the Butterworth filter, the decrease in SNR for both types of regions with the increase in cutoff frequency as compared to the Hamming filter was obtained. Overall, the Butterworth filter under investigation provided superior results than the Hamming filter. Effects of both filters on the quality of hot and cold region images varied with the change in cutoff frequency.

## 1. Introduction

In SPECT, image noise is an important factor may degrade the image quality. In clinical applications, image noise tends to limit the diagnostic accuracy and increases the difficulty in providing high quality medical services to patients. Compared to radiology, noise is more common in nuclear medicine imaging [1, 2] which can be reduced by using digital filters [3–6]. In this context, different types of filters, such as high-pass, low-pass, and band-pass, are used. For the enhancement of the image features in emission computed tomography, filters play an important role. However, there are several features that can be improved depending on the requirements of clinical examination [4–7]. In SPECT, filtering of the image data is done in order to suppress the noise signals. There are two categories of noise in nuclear medicine image data, namely, random noise and structured noise. Statistical variation in the count rate leads to the propagation of random noise which relates to the information density measured as counts per unit area. Presence of random noise in the image data affects the detectability of smaller sized regions and the contrast of the regions being studied. On the other hand, the structured noise that is described as the nonrandom inequalities in counting rate and distribution of radioactivity itself overlaid on which obscure the structural information of the organ of interest. For example, uptake of bowel in clinical SPECT to identify the inflammation with Ga-67 [8]. In addition, the structured noise may occur from imaging system artefacts, the nonuniformities in gamma camera images, such as ring or streak artefacts produced during the image reconstruction process which interferes with the visibility of the structure of the region of interest [8].

A wide variety of filtering algorithms including Ramp, Hamming, Chebyshev, Bessel, Butterworth, and Gaussian filters have been developed to remove noise from the image data. Filtering of the image data is one of the most common practices to produce high-quality images [9, 10]. It is used primarily to limit the effect of noise on image interpretation and analysis [7]. In addition, it is often useful to improve a specific feature, such as boundary detection of the region of interest [11, 12]. Filters can be applied in both spatial and frequency domains. Optimal filtration of a noisy image requires the preservation of low-frequency signals and the elimination of high-frequency signals. In the frequency domain, this type of filter could be described as a low-pass filter [3].

In this research, emphasis was given in two types of low-pass digital filters, namely, the Butterworth filter and Hamming filter used in FBP. These filters are the most commonly applied filters in clinical SPECT studies and are usually provided with the software of gamma cameras by manufacturers as default filters to the end users. However, these types of filters may cause loss of contrast.

The Butterworth filter used in FBP is a low-pass filter which is applied in SPECT in order to suppress high-frequency signals of noise. It is described by two parameters, the cutoff frequency and the order [13]. In the spatial frequency domain, the Butterworth filter is expressed by the following equation (4):

(1) $$\begin{array}{c} B\left(f\right)=\frac{1}{1+{\left(f/f_{\text{c}}\right)}^{2n}}, \end{array}$$

where *f* is the spatial frequency domain, *f*_c_ is the cutoff frequency, and *n* is the order of the filter.

Furthermore, the Hamming filter used in FBP is also a low-pass filter that is employed to reduce high-frequency signals of noise. The only parameter used to describe Hamming filter is its cutoff frequency [14]. Hamming filter is represented mathematically as [4]

(2) $$\begin{array}{c} H\left(f\right)=\left\{\begin{array}{cc} 0.54+0.46\;\cos\left(\frac{\pi f}{f_{m}}\right), & 0\leq \left|f\right|\leq f_{m},\\ 0, & \text{otherwise}, \end{array}\right. \end{array}$$

where *f* represents the spatial frequencies of the image and *f*_*m*_ is the corresponding cutoff frequency.

It is worth stating that in our research, the chosen parameters of both the filters, namely, cutoff frequency and order were different as compared to those selected in research published in [3–6]. In addition, a different type of phantom was scanned with the specific arrangements and positions of regions, particularly cold regions which were dissimilar relative to the phantoms scanned in previous studies [3–6]. The specific arrangement of hot and cold regions provided an opportunity to study and analyse the effects of both the filters simply and compare the results directly which could not be achieved by scanning the phantoms used in studies [3–6]. We compare the effects of both the filters on image quality in terms of hot and cold regions' detectability, contrast, and SNR selecting four different cutoff frequencies with a constant order.

## 2. Materials and Methods

### 2.1. Data Acquisition and Image Reconstruction

**Figure 1:**
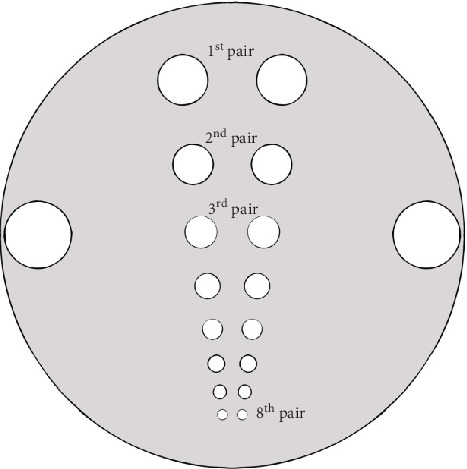
Cross-sectional view of hot regions' insert (cold regions' insert has similar view, not shown).

We employed an acrylic cylindrical phantom [15] with insert of holes drilled in a solid acrylic block mimicking the hot regions in a cold background (22.6, 18.1, 14.5, 11.6, 9.4, 7.5, 6.1, and 4.9 mm diameter), and solid circular rods simulating cold regions in a hot background with various diameters (22.5, 18.0, 14.4, 11.5, 9.3, 7.4, 6.0, and 4.8 mm), as shown in Figure 1. The advantage of this specific design provided similar transaxial views of hot and cold regions. Twenty (20) mCi of Tc-99m radionuclide was introduced into the phantom, the amount of radioactivity administered for most of clinical SPECT studies ranges 11 mCi–30 mCi. Data were acquired using the Philips ADAC forte dual head SPECT gamma camera mounted with a low-energy, high-resolution (LEHR) collimator. The phantom was placed on the patient's table at the center of the field of view of the gamma camera. A standard energy window (20%) centered at 140 keV was adjusted, and 128 × 128 matrix size was selected. Ninety views were taken over 360°, and the radius of rotation of gamma camera head was set at 35.7 cm. The time for each view selected was 20 seconds. Images were produced by filtered back projection image reconstruction technique using the Butterworth and the Hamming filters with different cutoff frequencies (0.35, 0.40, 0.45, and 0.50 cycles/cm), respectively. In terms of the filter order, a single value of order 7 for Butterworth filter was selected based on the research conducted by [4, 16, 17] which shows that filter order has less impact on the image quality. The Hamming filter utilizes a single parameter which is the cutoff frequency. Chang's method of attenuation correction with the value of linear coefficient 0.11/cm was applied [18]. Before the start of image reconstruction process, uniformity, center of rotation (COR), and radioactive decay corrections were applied on the data.

### 2.2. Image Quality Analysis

For image quality analysis, i.e., hot and cold regions' detectability, contrast, and SNR measurements, good quality transverse slices among others were selected. In this regard, three persons from the School of Health Sciences of one of the public universities with experience of nuclear medicine image analysis were requested. For hot and cold region analysis, 23^rd^ and 34^th^ cross-sectional slices were chosen, respectively. Hot and cold regions' detectability analysis was carried out by using ImageJ software [19]. Images reconstructed by applying both filters with different cutoff frequencies were uploaded in the ImageJ environment. Montage (multiple images displayed in one figure/window) of images consisting of both type of regions was separately obtained. For detectability of hot and cold regions, contour plotter function of ImageJ which identifies the contour automatically was applied by selecting the different count density values of regions of interest (ROIs), in order to view different sized regions.

Regarding the quantitative analysis, contrast and SNR of both (hot and cold) regions was measured by drawing ROIs using the Philips ADAC imaging system software provided by the vendor. A large irregular ROI for background/adjacent area count measurements was drawn carefully while avoiding overlapping of any hot and/or cold region. Average count density values were recorded for the calculation of contrast. The standard deviation (Sd) in the count density of hot and/or cold region (*D*_reg_) and the background/adjacent area (*D*_adj_) was recorded. In this analysis, for both regions (images obtained by applying the Butterworth filter), only four pairs were considered; the largest pair (hot and cold) at the edges of insert (opposite to each other at 180°) was excluded. However, in the case of the Hamming filter, three hot region pairs and only two cold region pairs were considered, whereas other pairs were not clearly visible. The hot region image contrast was obtained by using Equation (3) [20].

(3) $$\begin{array}{c} C_{\text{HR}}=\frac{D_{\text{reg}}-D_{\text{adj}}}{D_{\text{reg}}+D_{\text{adj}}}. \end{array}$$

In contrast, the cold region image contrast was obtained by applying Equation (4) [20].

(4) $$\begin{array}{c} C_{\text{CR}}=\frac{D_{\text{reg}}-D_{\text{adj}}}{D_{\text{adj}}}, \end{array}$$

where *D*_reg_ is the mean value of counts in the ROI of hot or cold region and *D*_adj_ is the mean value of counts in the ROI of adjacent area of hot or cold region.

The standard deviation in the contrast of hot and cold regions was calculated using Equations (5) and (6), respectively [21].

(5) $$\begin{array}{c} E_{\text{HRC}}=\text{sqrt}\left(\frac{\text{Sd}{D_{\text{reg}}}^{2}+\text{Sd}{D_{\text{adj}}}^{2}}{{\left(D_{\text{reg}}-D_{\text{adj}}\right)}^{2}}+\frac{\text{Sd}{D_{\text{reg}}}^{2}+\text{Sd}{D_{\text{adj}}}^{2}}{{\left(D_{\text{reg}}+D_{\text{adj}}\right)}^{2}}\right), \end{array}$$

(6) $$\begin{array}{c} E_{\text{CRC}}=\text{sqrt }\left(\frac{\text{Sd}{D_{\text{reg}}}^{2}+\text{Sd}{D_{\text{adj}}}^{2}}{{\left(D_{\text{reg}}-D_{\text{adj}}\right)}^{2}}+\frac{\text{Sd}{D_{\text{adj}}}^{2}}{{D_{\text{adj}}}^{2}}\right), \end{array}$$

where the HRC and CRC represent the hot and cold region image contrast, respectively. Sd*D*_reg_ is the standard deviation in *D*_reg_, and Sd*D*_adj_ is the standard deviation in *D*_adj_.

The percentage decrease or increase in the image contrast of hot regions of different sizes (22.6, 18.1, 14.5, and 11.6 mm diameter) and cold regions of different sizes (22.5, 18.0, and 14.4 mm diameter) with the Butterworth filter by selecting different cutoff frequencies as mentioned above was calculated using Equation (7) [22]. The same equation was applied for the calculation of percent decrease or increase in the contrast of hot regions (22.6, 18.1, and 14.5 mm diameter) and cold regions of 22.5 and 18.0 mm diameter with the Hamming filter with a 0.35, 0.40, 0.45, and 0.50 cycles/cm cutoff frequency.

(7) $$\begin{array}{c} \text{Percentage increase or decrease}=\frac{\left(V_{2}-V_{1}\right)}{\left|V_{1}\right|}\times 100, \end{array}$$

where *V*_2_ is the contrast value of a region (hot or cold) obtained by a cutoff frequency of the filter (the Butterworth or the Hamming) and *V*_1_ is the calculated contrast value of the same hot or cold region with a different cutoff frequency of the Butterworth filter or the Hamming filter.

Moreover, the percentage increase or decrease in the contrast of a hot or cold region (that included for analysis) was compared with the average values of contrast obtained by applying a 0.35 and 0.45 cycles/cm cutoff frequency with the mean contrast value of the same sized hot or cold region that was calculated by using 0.40 and 0.45 cycles/cm cutoff frequency of the Butterworth filter or the Hamming filter.

The signal-to-noise ratio of hot and cold region was calculated by Equations (8) and (9), respectively [20].

(8) $$\begin{array}{c} {\text{SNR}}_{\text{HR}}=\frac{\left|D_{\text{reg}}-D_{\text{adj}}\right|}{\sqrt{{\text{Sd}D}_{{\text{reg}}^{2}}+{\text{Sd}D}_{{\text{adj}}^{2}}}}, \end{array}$$

(9) $$\begin{array}{c} {\text{SNR}}_{\text{CR}}=\frac{\left|D_{\text{reg}}-D_{\text{adj}}\right|}{\sqrt{\text{Sd}{D_{\text{adj}}}^{2}}}. \end{array}$$

Percentage decrease in the SNR of hot and cold regions was calculated using Equation (7) [22], where *V*_1_ is the SNR value of a hot or cold region obtained by a cutoff frequency of the Butterworth or the Hamming filter and *V*_2_ is the SNR value of the same hot or cold region with a different cutoff frequency of the Butterworth filter or the Hamming filter.

The SPSS software was used for calculation of standard deviation in SNR. For this purpose, each measurement was repeated three times. The significance (*p* < 0.05) of results was performed by one-way ANOVA.

## 3. Results

**Figure 2:**
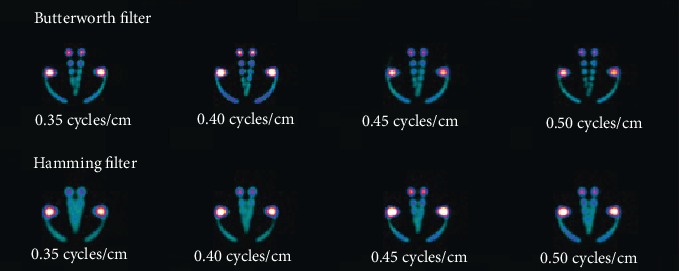
Hot region images obtained by selecting various cutoff frequencies of the Butterworth filter (top row) and the Hamming filter (bottom row).

**Figure 3:**
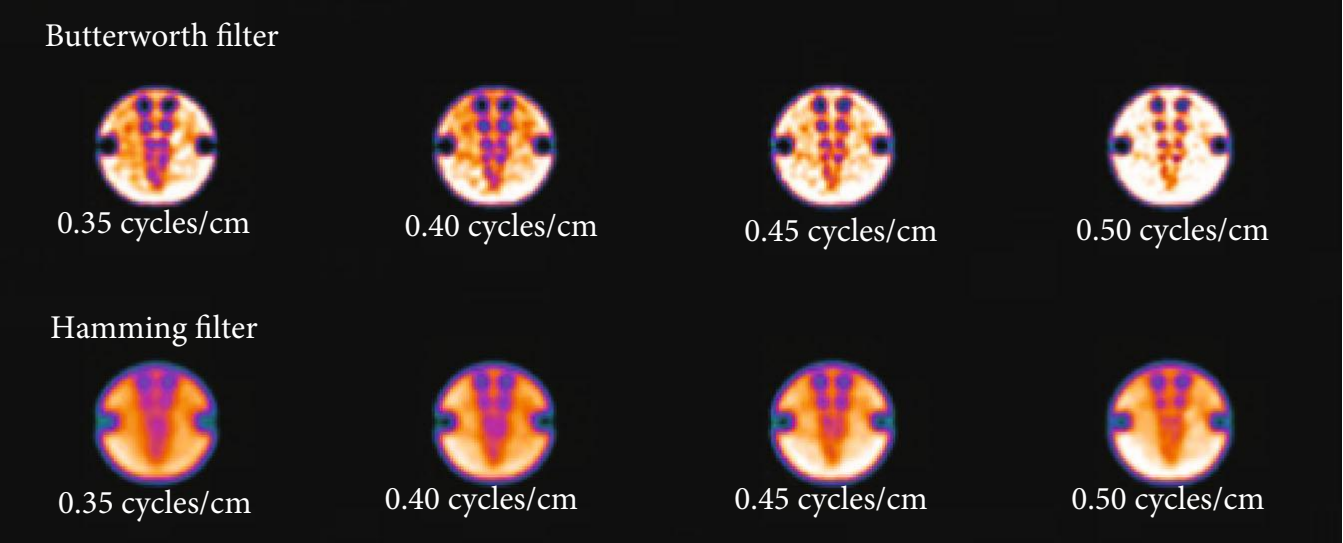
Cold region images obtained by selecting various cutoff frequencies of the Butterworth filter (top row) and the Hamming filter (bottom row).

In this research, emphasis was given on the investigations into the effects on hot and cold regions image quality of two different types of reconstruction filters, i.e., the Butterworth and the Hamming. These mathematical filters are categorized as passive filters which allow low frequencies to pass and are most commonly used in SPECT hot and cold region images used for analysis are shown in Figures 2 and 3, respectively.

### 3.1. Hot Regions' Detectability, Contrast, and SNR

#### 3.1.1. Butterworth Filter

**Figure 4:**
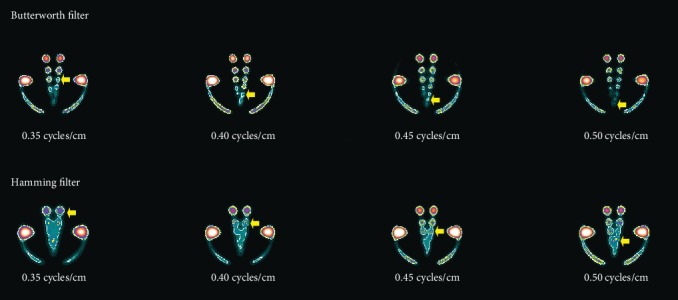
Contours of hot regions with the Butterworth filter (top row) and with the Hamming filter (bottom row).

Hot region images as shown in Figure 4 were visually inspected. Four pairs of hot regions (22.6, 18.1, 14.5, and 11.6 mm diameter) out of eight pairs (top row) with the use of all cutoff frequencies can be clearly seen.

However, with the increase in cutoff frequency, particularly with 0.45 and 0.50 cycles/cm, more pairs of hot regions were detected as indicated by the yellow arrows in Figure 4. In addition, higher cutoff frequency values (0.45 and 0.5 cycles/cm) enhanced edges of hot regions by removing background noise at the cost of distortion in the shape.

Image contrast is usually expressed as the regional change in photon density in a target area to that in an adjacent area. For spherical region (approximately the size of system's spatial resolution) to be detected in a uniform adjacent area, the difference between the density of two regions must be greater than about 0.07 [23]. The worst and better contrast value can be distinguished using the scale of 0 to 1; the value nearer to 0 is considered worst and closer to 1 better.

Measured contrast values of four hot regions of various sizes, presented in Table 1, show the decrease in the contrast of hot regions images reconstructed by applying 0.45 and 0.50 cycles/cm compared to the 0.35 and 0.40 cycles/cm cutoff frequency. The decrease in the contrast was measured, 16% for 22.6 mm, 19% for 18.1 mm and 14.5 mm, and 23% for 11.6 mm diameter hot region, approximately.

**Figure 5:**
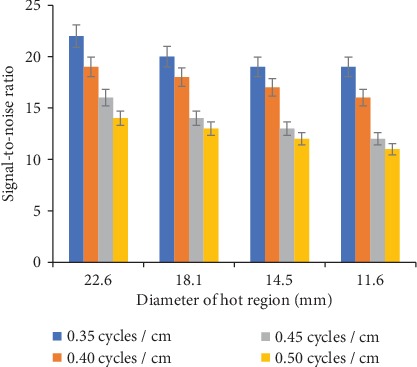
Hot regions SNR with various cutoff frequency values of the Butterworth filter.

Figure 5 shows the SNR of hot regions of different diameters with different cutoff frequency values. A significant decrease (≈37-42%) in SNR for all sizes of hot regions with the increase in cutoff frequency (0.35-0.50 cycles/cm) was recorded. However, a significant but relatively less decrease (≈14-25%) in SNR with respect to the size of each hot region with a single cutoff frequency, for example, 22.6, 18.1, 14.5, and 11.6 mm diameter, with a 0.35 cycles/cm cutoff frequency was observed.

#### 3.1.2. Hamming Filter

In the bottom row of images of Figure 4, only one pair of hot regions at a 0.35 cycles/cm cutoff frequency can be seen clearly, namely, the one with the 22.6 mm diameter. With the use of 0.40 cycles/cm cutoff frequency, two pairs of hot regions were detectable (22.6 mm and 18.1 mm diameter). However, with other cutoff frequency values (0.45 and 0.50 cycles/cm), three pairs of hot regions were visible. In addition, the contours pinpointed by yellow arrows in Figure 4 investigated the location of the fourth pair when 0.50 cycles/cm cutoff frequency was used on the image data during the reconstruction process.

For the measurement of contrast, only one pair of hot regions of diameter 22.6 mm for 0.35 cycles/cm cutoff frequency, two pairs of hot regions (22.6 mm and 18.1 mm diameter) for 0.40 cycles/cm cutoff frequency, and three pairs of hot regions (22.6, 18.1, and 14.5 mm diameter) for 0.45 and 0.50 cycles/cm cutoff frequency were included as shown in Table 2. Improvement in the contrast of 22.6 mm diameter hot region has been recorded by increasing the cutoff frequency. Enhancement in the contrast with the increase in cutoff frequency was calculated as 9-32% only for 22.6 mm diameter hot region, and for other hot regions, overall equivocal contrast values were measured.

Comparison of hot regions results for both filters show enhancement in detectability with the increase in cutoff frequency. The Butterworth filter provided higher contrast values, compared to the Hamming filter. However, for a 0.45 and 0.50 cycles/cm cutoff frequency, a decrease in the contrast was noted. Conversely, an increase in the cutoff frequency of the Hamming filter enhanced the contrast of 22.6 mm diameter hot region, whereas no significant change in the contrast of 18.1 and 14.5 mm diameter hot regions was observed.

**Figure 6:**
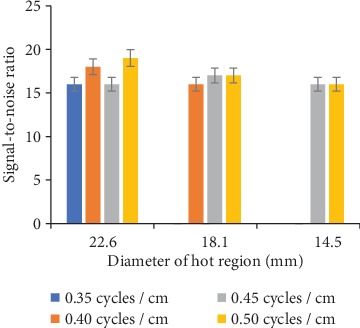
Hot regions SNR with various cutoff frequency values of the Hamming filter.

Signal-to-noise ratio of hot regions of various sizes with the Hamming filter of different cutoff frequency is shown in Figure 6. The increase (≈12-19%) in SNR only for 22.6 mm diameter hot region with 0.40 and 0.50 cycles/cm was recorded. However, almost no change in the SNR for hot regions of 18.1 and 14.5 mm diameter with the increase in cutoff frequency was measured.

In comparison, the SNR for hot regions calculated with both the filters selecting various cutoff frequency values show that the Butterworth filter provided a significant decrease in SNR as compared to the Hamming filter with the increase in cutoff frequency. On the other hand, with the increase in cutoff frequency of the Hamming filter, an increase in SNR relative to the Butterworth filter was noticed. In addition, higher SNR values with the Hamming filter were recorded for 0.45 and 0.50 cycles/cm cutoff frequency as compared to the Butterworth filter.

### 3.2. Cold Regions' Detectability, Contrast, and SNR

#### 3.2.1. Butterworth Filter

**Figure 7:**
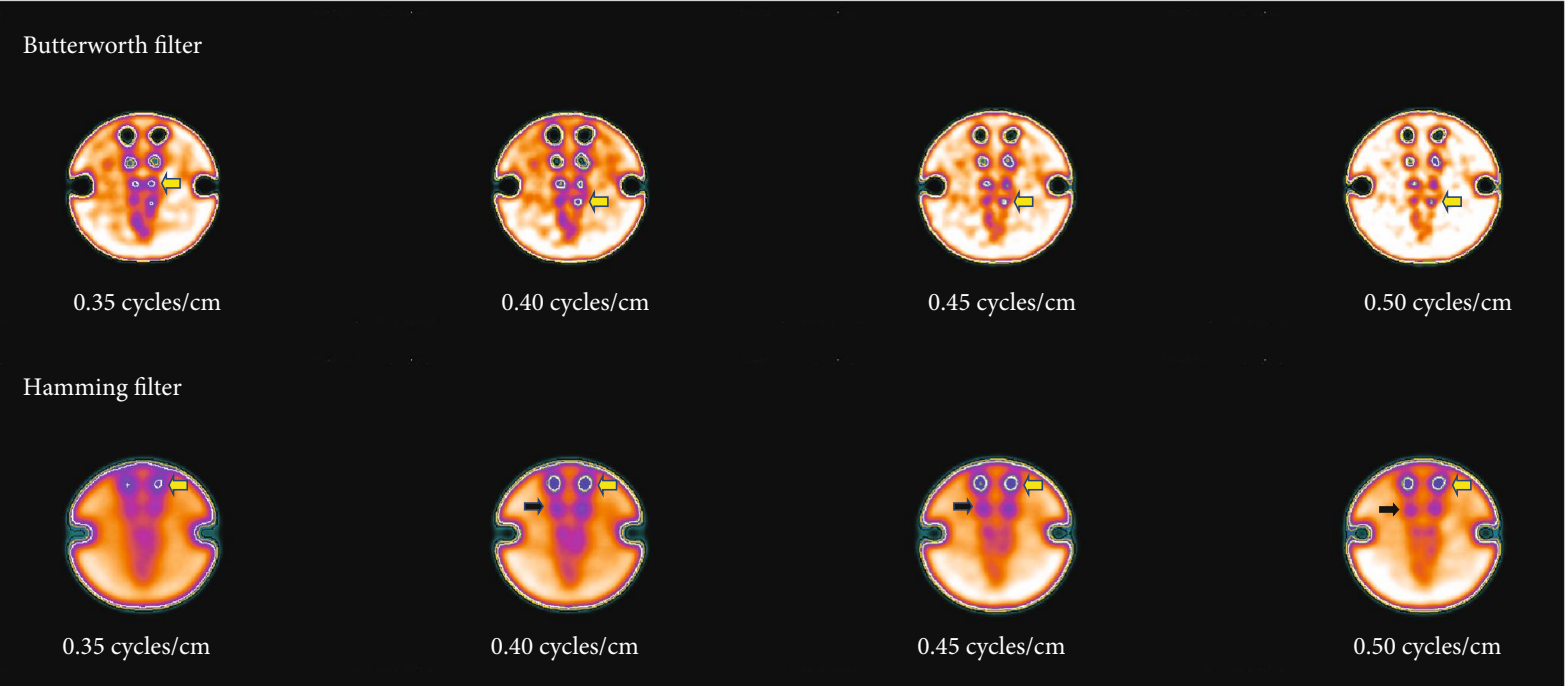
Contours of cold regions with the Butterworth filter (top row) and with the Hamming filter (bottom row).

In Figure 7, all images (top row) of cold regions show clearly three cold region pairs (22.5, 18.0, and 14.4 mm diameter) which were obtained by using cutoff frequencies of 0.35, 0.40, 0.45, and 0.50 cycles/cm, respectively.

Furthermore, one cold region of the fourth pair (11.5 mm diameter) is also visible. However, contour indicated by the arrow of yellow colour in images depict distorted shapes of smaller cold regions, compared to larger cold regions. It is worth noting that in the phantom used, all regions are circular in shape.

Cold regions' contrast is shown in Table 3. A 0.35 cycles/cm cutoff frequency provided higher contrast values for all regions except 22.5 mm diameter cold region. Overall, with the increase in cutoff frequency (0.35 to 0.50 cycles/cm with the step of 0.05 cycles/cm), a decrease in the contrast of all cold regions was observed. Moreover, the smaller the cold region, the higher the decrease in the contrast, approximately 15% for 22.5 mm, 50% for 18.0 mm, 61% for 14.4 mm, and 82% for 11.5 mm diameter cold region.

**Figure 8:**
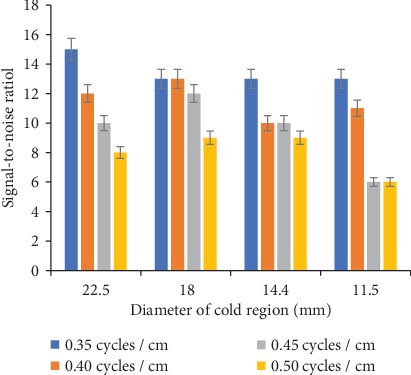
Cold regions SNR with various cutoff frequency values of the Butterworth filter.

In Figure 8, SNR of cold regions of various diameter with the Butterworth filter of different cutoff frequency is presented. For larger cold regions (22.5 and 18 mm diameter), a significant decrease (≈47 and 30%, respectively) in the SNR with the increase in cutoff frequency, particularly with a 0.45 and 0.50 cycles/cm cutoff frequency, was noticed. However, for 14.4 and 11.5 mm diameter cold regions, SNR decreased (≈30 and 54%, respectively) with the increase in cutoff frequency.

#### 3.2.2. Hamming Filter

In the cold region images depicted in Figure 7 (bottom row), one pair (22.5 mm diameter) is visible at a 0.35 cycles/cm cutoff frequency. Two pairs of cold regions, 22.5 and 18.0 mm diameter (only the location of 2^nd^ pair -18.0 mm diameter indicated by a black arrow), can be observed when 0.40, 0.45, and 0.50 cycles/cm cutoff frequency was applied. Furthermore, the remaining cold region pairs were undetectable for all cutoff frequencies.

In terms of the contrast of cold regions, overall improvement is observed with the increase in cutoff frequency. However, contrast values are low as shown in Table 4. The percentage increase in the contrast was about 20% for 22.5 mm, and the equivocal contrast values for the 18.0 mm diameter cold regions were obtained.

**Figure 9:**
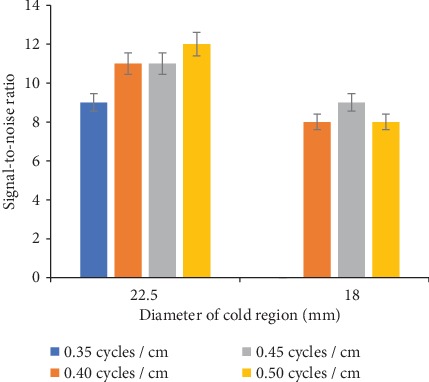
Cold regions SNR with various cutoff frequency values of the Hamming filter.

The signal-to-noise ratio of two cold regions (22.5 and 18 mm diameter) calculated from images obtained using the Hamming filter with four different cutoff frequencies is shown in Figure 9. About 33% increase in SNR of 22.5 mm diameter cold region with the increase in cutoff frequency was recorded. For 18 mm sized (diameter) cold region, approximately 11% increase in SNR was calculated with a 0.45 cycles/cm cutoff frequency, whereas with a 0.40 and 0.50 cycles/cm cutoff frequency, no change in SNR was obtained.

The SNR of all cold regions with the Butterworth filter decreased with the increase in the cutoff frequency. However, the Hamming filter showed increase in SNR for larger cold region (22.5 mm) with the increase in cutoff frequency, whereas increase in SNR of 18 mm diameter cold region was noticed only at 0.45 cycles/cm cutoff frequency.

## 4. Discussion

In SPECT, the use of digital filters is imperative for removing noise from the image data. The presence of noise leads to the poor quality of reconstructed images which in turn hinder the accuracy of the diagnosis. There exist many digital filters; however, use of a single filter with selected parameters on the data of different types of clinical studies is impractical. In general, selection of digital filters and their appropriate parameters for the use on the clinical SPECT data is based on the operator's observations and experience instead of theoretical or logical scientific justification [4, 24]. However, the choice of a filter and its functions for a particular image processing assignment is a balancing act between noise removal, enhancement of image details, and contrast [25]. The corresponding special frequency pattern of the image data in question. In this study, the effects of the Butterworth and the Hamming filters used in FBP by changing cutoff frequency on the SPECT image quality of hot and cold regions were investigated. Results of both filters were compared. The range of the order of the Butterworth filter commonly used in clinical examination is 5–10. Despite that, the order 7 was selected because higher order may produce the rippling artefact in the reconstructed image [1].

The results of our study indicate that the Butterworth filter enhanced the detectability of hot regions at the cost of the distortion of smaller hot regions when higher cutoff frequency values were chosen, such as 0.45 and 0.50 cycles/cm. In clinical conditions, this would affect the texture of the organ in the resulting image which in turn could lead to the inaccuracy in the diagnosis of the disease. The Butterworth filter efficiently removed the noise which resulted in the enhancement in image details. Furthermore, it also indicated that the impact on image quality varies with respect to the size of hot regions as well as the count density in the region of interest which is the case in this study. Moreover, higher frequency corresponds to small regions and to abrupt changes in the count density of regions of interest [26–29].

On the other hand, with the Hamming filter, we were able to see clearly only two pairs of hot regions (out of eight pairs at 0.35 and 0.40 cycles/cm cutoff frequency), whereas the remaining hot regions were undetectable. However, with higher cutoff frequency, the image revealed two more pairs. Furthermore, our results depicted the smoother background which verifies that this filter is a high-smoothing filter, hence the loss in image detail and the degradation of contrast [4, 26].

As far as the contrast of hot and cold regions is concerned, the Butterworth filter showed the decrease in the contrast of all sizes of hot and cold regions when higher cutoff frequencies (0.45 and 0.50 cycles/cm) were applied as compared to 0.35 and 0.40 cycles/cm cutoff frequency as shown in Tables 1 and 3, respectively. The Hamming filter at a 0.35 cycles/cm cutoff frequency for 22.6 mm diameter hot region showed improvement; however, no change in the contrast was observed with the increase in cutoff frequency for smaller hot regions.

Detectability results of cold regions using the Butterworth filter could expose clearly only three pairs of cold regions out of eight pairs. One region of the fourth pair was detectable at 0.50 cycles/cm cutoff frequency. However, smaller regions suffered the distortion in terms of shape. The Hamming filter could only show one pair at 0.35 cycles/cm cutoff frequency, and with the use of other frequencies, two pairs were visible, whereas the remaining regions were undetectable. The contrast of cold regions provided by the Butterworth filter decreased with the increase in cutoff frequency. Conversely, with the Hamming filter, the contrast of the largest cold region was higher as opposed to other smaller regions, where almost no change was observed.

Besides the visual inspection and contrast measurement, SNR is one of the indicators among others used for evaluation of SPECT image quality, where the strength of signal as compared to the noise in an image is determined [30]. Usually, for fair and easy detection of region/lesion from its background, the SNR value of 3 to 5 is required [8].

The SNR results of hot regions showed that the Butterworth filter provided a significant decrease in SNR with the increase in cutoff frequency. This is due to the increase in cutoff frequency attenuate the high-frequency signals which enhances the noise in an image that leads to the decrease in SNR. The lower value of SNR of smaller hot regions as compared to the larger hot regions were recorded. This is because SNR is count statistics, region/lesion size, and depth sensitive [31, 32]. Contrary, with the increase in cutoff frequency of the Hamming filter, an increase in SNR of 22.6 mm diameter hot region at 0.40 and 0.50 cycles/cm cutoff frequency was achieved. This could be due to its high degree smoothing factor [4]. However, no change in the result for 18 and 14.5 mm diameter hot regions was noted.

The Butterworth filter presented the decrease in SNR of all cold regions with the increase in cutoff frequency. Conversely, the Hamming filter showed increase in SNR for larger cold region (22.5 mm) with the increase in cutoff frequency. For 18 mm cold region, a significant increase in SNR was noticed only at 0.45 cycles/cm cutoff frequency. The reasons for the decrease and increase in SNR with the Butterworth filter and the Hamming filter, respectively, could be the same as the reasons for the hot region.

Overall, the Butterworth filter resulted better detectability and contrast of hot and cold regions than the one of the Hamming filter, more hot region pairs were detected as opposed to cold regions. Regarding the contrast, higher values of hot regions were recorded compared to the contrast of cold regions. However, the Butterworth filter shows the decrease in SNR with the increase in cutoff frequency compared to the Hamming filter. Our results show different effects of cutoff frequency of both filters on hot and cold regions.

## 5. Conclusions

The Butterworth filter results were superior except SNR to those obtained by the application of the Hamming filter. Therefore, the Butterworth filter has proved to be a better filter than the Hamming filter. However, the effects of both filters by changing the cutoff frequency differ from one another. When the Butterworth filter must be applied in clinical data, especially taking into account the type of the study and the corresponding ROIs, it is important to choose the correct cutoff frequency prior to the image reconstruction with respect to the type of study and expected regions of interest (hot and cold).

## Figures and Tables

**Table 1:** Hot regions' contrast with various cutoff frequency values of the Butterworth filter.

Cutoff frequency (cycles/cm)	Hot regions' contrast			
	22.6 mm	18.1 mm	14.5 mm	11.6 mm
0.35	0.89 ± 0.06	0.87 ± 0.06	0.86 ± 0.07	0.86 ± 0.07
0.40	0.90 ± 0.07	0.89 ± 0.07	0.88 ± 0.07	0.87 ± 0.08
0.45	0.76 ± 0.08	0.72 ± 0.08	0.70 ± 0.08	0.67 ± 0.09
0.50	0.76 ± 0.09	0.73 ± 0.09	0.71 ± 0.09	0.68 ± 0.10

**Table 2:** Hot regions' contrast with various cutoff frequency values of the Hamming filter.

Cutoff frequency (cycles/cm)	Hot regions' contrast		
	22.6 mm	18.1 mm	14.5 mm
0.35	0.39 ± 0.07	—	—
0.40	0.48 ± 0.07	0.60 ± 0.06	—
0.45	0.51 ± 0.07	0.62 ± 0.06	0.61 ± 0.06
0.50	0.57 ± 0.06	0.60 ± 0.06	0.58 ± 0.06

**Table 3:** Cold regions' contrast with various cutoff frequency values of the Butterworth filter.

Cutoff frequency (cycles/cm)	Cold regions' contrast (-)			
	22.5 mm	18.0 mm	14.4 mm	11.5 mm
0.35	0.45 ± 0.19	0.67 ± 0.05	0.74 ± 0.05	0.95 ± 0.05
0.40	0.46 ± 0.16	0.49 ± 0.06	0.55 ± 0.06	0.85 ± 0.06
0.45	0.44 ± 0.11	0.30 ± 0.05	0.29 ± 0.05	0.16 ± 0.05
0.50	0.39 ± 0.13	0.31 ± 0.06	0.29 ± 0.06	0.17 ± 0.06

**Table 4:** Cold regions' contrast with various cutoff frequency values of the Hamming filter.

Cutoff frequency (cycles/cm)	Cold regions' contrast (-)	
	22.5 mm	18.0 mm
0.35	0.28 ± 0.11	—
0.40	0.33 ± 0.09	0.24 ± 0.10
0.45	0.31 ± 0.10	0.25 ± 0.11
0.50	0.35 ± 0.08	0.24 ± 0.09

## Data Availability

Supporting data are included in research article.
